# Subtypes of asthma based on asthma control and severity: a latent class analysis

**DOI:** 10.1186/s12931-017-0508-y

**Published:** 2017-01-23

**Authors:** Elina M. S. Mäkikyrö, Maritta S. Jaakkola, Jouni J. K. Jaakkola

**Affiliations:** 1 Center for Environmental and Respiratory Health Research (CERH), University of Oulu, PO Box 5000, FI-90014 Oulu, Finland; 2 Medical Research Center Oulu (MRC Oulu), Oulu, Finland

**Keywords:** Asthma, Epidemiologic study, Latent class analysis, Asthma subtypes, Asthma control, Asthma severity, Determinant, Risk factor

## Abstract

**Background:**

Asthma subtyping is a complex new field of study. Usually both etiological and outcome factors of asthma have been used simultaneously for subtyping thus making the interpretation of the results difficult. Identification of subtypes of asthma based on questionnaire data only will be useful for both treatment of asthma and for research. Our objective was to identify asthma subtypes that capture both asthma control and severity based on easily accessible variables.

**Methods:**

We applied latent class analysis for the 1995 adult asthmatics, 692 men and 1303 women, of the Northern Finnish Asthma Study (NoFAS). The classifying variables included use of asthma medication within the last 12 months, St. George’s Respiratory Questionnaire score, and asthma-related healthcare use within the last 12 months. Covariates adjusted for included COPD, allergic rhinitis/allergic eczema, BMI, age and sex. All information was based on self-administered questionnaires.

**Results:**

We identified four subtypes for women: *Controlled, mild asthma* (41% of participants); *Partly controlled, moderate asthma* (24%); *Uncontrolled asthma, unknown severity* (26%), and *Uncontrolled, severe asthma* (9%). For men we identified three subtypes: *Controlled, mild asthma* (31%); *Poorly controlled asthma, unknown severity* (53%); and *Partly controlled, severe asthma* (17%). For almost 96% of the subjects this subtyping was accurate. The covariates fitted in the model were based on clinical judgment and were good predictors of class membership.

**Conclusions:**

Our results show that it is possible to form meaningful and accurate asthma subtypes based on questionnaire data, and that separate classification should be applied for men and women.

**Electronic supplementary material:**

The online version of this article (doi:10.1186/s12931-017-0508-y) contains supplementary material, which is available to authorized users.

## Background

Recently, multiple studies have applied clustering methods to form asthma and wheeze subtypes [1–12]. In the previous literature, both etiological and outcome factors of asthma have been included as classification variables thus complicating further analyses on e.g. risk factors of these subtypes. Also, subtypes formed so far have included several clinical markers not always available in primary health care or large epidemiological studies. Thus, alternative subtyping is needed to benefit these purposes. It has also been commented, that the selection of variables in the previous studies has been wide and diverse [13]. In clinical practice, the focus is usually on two aspects of asthma: asthma severity and control.

Asthma control has been assessed by using self-administered questionnaires, information on asthma related healthcare use, or a priori grouping of individuals [14–17]. Determinants of poor asthma control identified previously include smoking [18], obesity [19], concomitant diseases such as rhinitis and COPD [20, 21], gender and age [22]. Poor asthma control affects the quality of life of asthma patients and increases the burden on the health care system [23, 24]. Asthma severity is another aspect applied for clinical evaluation of asthma patients. It consists partly of similar features as asthma control, but also has its’ independent dimension [25]. The ATS/ERS guidelines described severity as “difficulty of controlling asthma with adequate treatment” [25]. At current state of knowledge, the key questions for further research include 1) to what extent the interrelated aspects of asthma control and severity can be separated, and 2) how such separation should influence the treatment, care, and prognosis of asthma patients.

To unravel the complex links and differences between asthma control and severity, we studied a total of 1995 adult asthmatics from the Northern Finnish Asthma Study by applying latent class analysis (LCA) [26, 27]. The purpose of this study was to identify subtypes of asthma based on questionnaire-data only, among subjects already diagnosed to have asthma based on the diagnostic criteria applied in Finland. In order to enable further analyses on etiological factors of the subtypes formed, we included only variables of asthma manifestations in the classification. This enables further risk-factor analyses by maintaining the traditional idea of causality. The subtypes identified in this study can be applied in studies investigating potential role of environmental and behavioral factors in determining the etiology and/or prognosis (of different subtypes) of asthma. We addressed the following questions: 1) Is it possible to identify asthma subtypes which characterize the aspects of both asthma control and asthma severity by applying questionnaire-based information only; 2) When we assign a person to a particular subtype, how certain can we be that he/she truly belongs to that subtype; and 3) Do the subtypes formed separately for men and women lead to a more accurate classification? We also assessed 4) whether the following characteristics: age, having COPD and/or allergic diseases, and BMI, predict belonging to a certain asthma subtype.

## Methods

### Study population

The Northern Finnish Asthma Study (NoFAS) was initiated in 2012 as a population-based cross-sectional study of adults 17–73 years old who had asthma and who lived in Northern Finland. The source population constituted of subjects who had received the reimbursement right for asthma medication, thus fulfilling the diagnostic criteria of The Social Insurance Institution of Finland. The subjects lived in The Oulu University Hospital District. We sent two self-administrated questionnaires, the NoFAS respiratory questionnaire and the St George’s Respiratory Questionnaire to a random sample of 5000 subjects. A total of 2033 (40.7%) subjects responded. However, we excluded respondents whose age and/or sex were unknown, so the final study population included 1995 subjects (response rate 40%). The data collection has been described in detail in a previous study [28].

### Variables used in the latent class analysis

We applied the following variables in the classification of asthma: use of controller asthma medication, bronchodilators, oral corticosteroids, and/or antibiotics during asthma exacerbations, and use of various healthcare services. All of these were inquired for the past 12 months. In addition, St. George’s Respiratory Questionnaire score in the past 4 weeks was applied.

Controller asthma medication was defined by the use of inhaled corticosteroids (ICS) based on replies to the questions: “During the previous year, have you used inhaled steroids?” and/or “During the previous year, have you used inhaled combination medication of corticosteroid and long-acting bronchodilator?” Inhaled bronchodilator (BD) use was defined by reply to the question: “During the past year, have you used inhaled bronchodilator?” Oral corticosteroid use (OCS) was defined by combining responses to two questions: “During the past year, have you used oral corticosteroid tablets” and “During the past year, have you received prescription for oral corticosteroids? If yes, how many courses?” Use of antibiotics for asthma exacerbations (AB) was defined based on the following question: “During the last 12 months, have you been prescribed antibiotics for asthma symptoms? If yes, how many courses?”

**Fig. 1:**
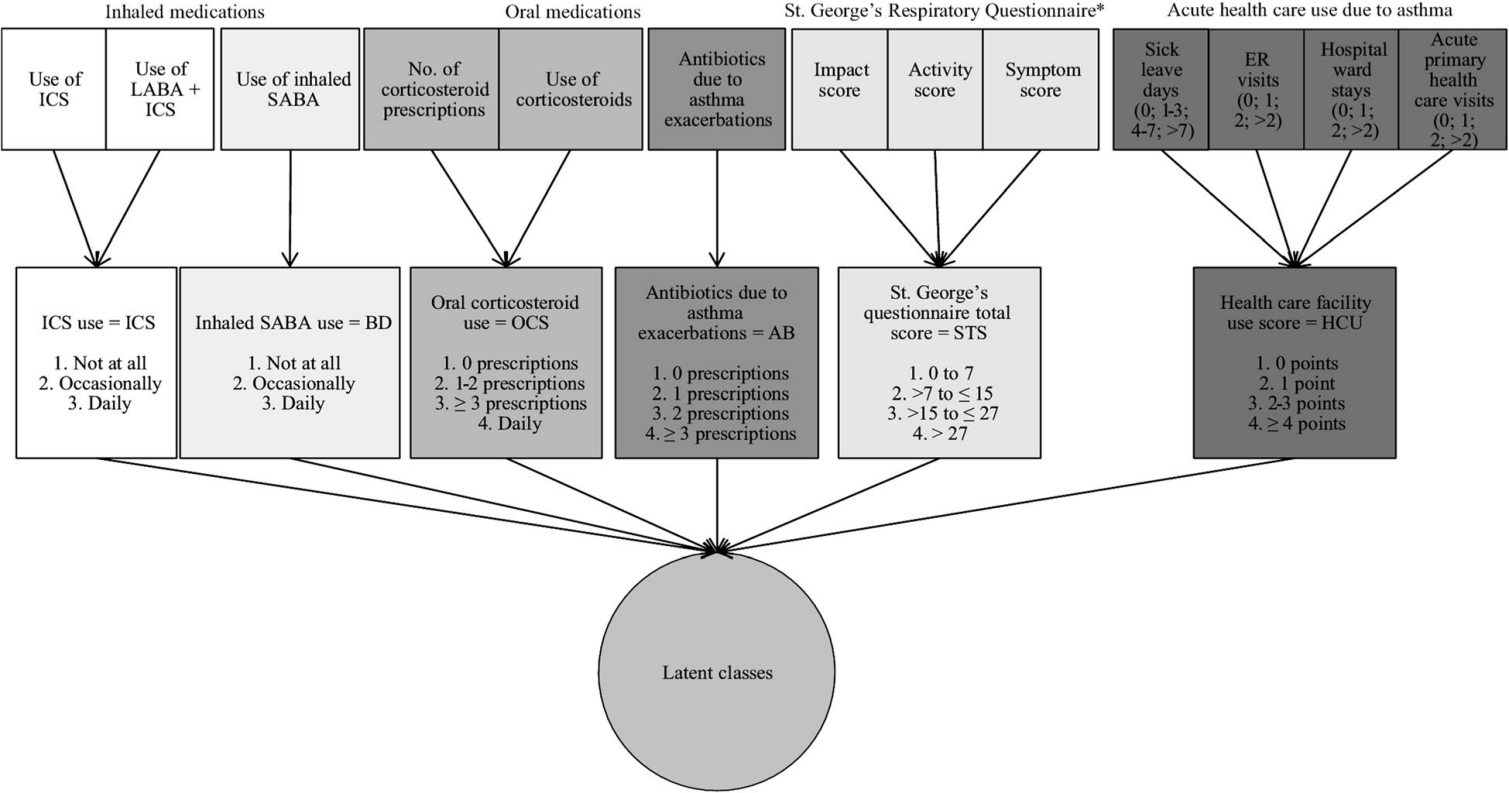
Variable selection and the combination of variables used in forming latent classes. The upper level indicates the types of variables directly derived from the questionnaire. The second level describes the combinations formed based on those variables and the levels used for latent class forming. Altogether six variables were included in the classification

The St. George’s Respiratory Questionnaire total score (SGTS) was calculated for the period of the past 4 weeks and a categorical variable was created so that the baseline level corresponded to the scores detected in healthy subjects (i.e. 0–7 points) [29, 30]. SGTS has been reported in previous studies to correlate with the duration of asthma symptoms, the level of lung function measurements, and the history of asthma exacerbations [31]. The healthcare use score (HCU) was defined as the sum of the number of 1) sick leave days, 2) emergency room visits, 3) hospital ward treatments, and 4) acute primary health care visits, all due to asthma during the previous 12 months. Any health care visit because of acute asthma exacerbation is nowadays rare in Finland, and thus, high HCU scores were rare in our study population [32]. The variables that were used in the analyses, and their prevalence in the whole study population as well as in men and women separately, are presented in Table 1. Figure 1 displays the original variables in the questionnaires and the pathway that led to the finally defined categorical variables of asthma control and severity.

**Table 1:** Characteristics of the study population

	Women	Men	Total population
	*N* (%)	*N* (%)	*N* (%)
Total *N*	1303 (65.3)	692 (34.7)	1995 (100.0)
Age			
<30 years	141 (10.8)	71 (10.3)	212 (10.6)
30–59 years	848 (65.1)	420 (61.7)	1268 (63.6)
≥60 years	314 (24.1)	201 (29.1)	515 (25.8)
Asthma controller medication use^a^			
Not at all	80 (6.2)	48 (7.1)	128 (6.5)
Occasionally	200 (15.6)	103 (15.2)	303 (15.4)
Daily	1006 (78.2)	528 (77.8)	1534 (78.1)
Missing	17	13	30
Inhaled bronchodilatator use			
Not at all	217 (17.2)	147 (22.0)	364 (18.9)
Occasionally	834 (66.0)	365 (54.7)	1199 (62.1)
Daily	213 (16.9)	155 (23.2)	368 (19.1)
Missing	39	25	64
Antibiotics for asthma exacerbations			
0 prescription	970 (76.1)	590 (87.0)	1560 (79.9)
1 prescription	119 (9.3)	41 (6.1)	160 (8.2)
2 prescriptions	80 (6.3)	23 (3.4)	103 (5.3)
≥3 prescriptions	106 (8.1)	24 (3.5)	130 (6.7)
Missing	28	14	42
Oral corticosteroid use			
0 prescriptions	893 (70.5)	558 (83.2)	1451 (74.9)
1–2 prescriptions	294 (23.2)	86 (12.8)	380 (19.6)
≥3 prescriptions	49 (3.9)	20 (3.0)	69 (3.6)
Daily use	30 (2.4)	7 (1.0)	37 (1.9)
Missing	37	21	58
St. George’s score 4 weeks^b^			
0 to ≤7	300 (23.3)	180 (26.6)	480 (24.4)
>7 to ≤15	327 (25.4)	142 (21.0)	469 (23.9)
>15 to ≤27	331 (25.7)	166 (24.5)	497 (25.3)
>27	330 (25.6)	189 (27.9)	519 (26.4)
Missing	15	15	30
Health care facility use score^c^			
0 points	936 (73.0)	580 (84.2)	1516 (76.9)
1 point	107 (8.4)	34 (4.9)	141 (7.2)
2–3 points	183 (14.3)	56 (8.1)	239 (12.1)
≥4 points	56 (4.4)	19 (2.8)	75 (3.8)
Missing	21	3	24
COPD diagnosis			
Yes	126 (10.0)	152 (23.2)	278 (14.5)
No	1135 (90.0)	504 (76.8)	1639 (85.5)
Missing	42	36	78
Allergic Rhinitis and/or Allergic Eczema			
Yes	891 (68.9)	402 (59.4)	1293 (65.9)
No	394 (30.7)	275 (40.6)	669 (34.1)
Missing	18	15	33
BMI			
≤ 25	504 (39.6)	216 (31.6)	720 (36.8)
>25 to ≤30	420 (33.0)	286 (41.8)	706 (36.1)
>30	350 (26.9)	170 (26.3)	532 (26.7)
Missing	29	8	37

^a^Asthma controller medication use includes both inhaled corticosteroids and combination medication of inhaled corticosteroid and long-acting beta-agonist

^b^St. George’s score range: 0–100

^c^HCU score formed as a combination of sick leave due to asthma, emergency room visits for asthma, ward treatment periods for asthma, and acute primary health care visits due to asthma

### Statistical methods

We applied latent class analysis (LCA), a method used to classify observations into discrete, mutually exclusive classes on the basis of categorical manifest variables [26, 27]. We formed classes based on the whole study population of 1995 subjects, and conducted subtypes analyses by gender, as the test for measurement invariance implicated that this was advisable [27]. We then fitted the following covariates into the model: diagnosis of COPD [21], allergic rhinitis and/or allergic eczema [20], BMI [19], age, and gender [22]. These covariates are known or suggested determinants of poor asthma control and severity. We also examined the clinical relevance of the identified subtypes by calculating the likelihood of a person belonging to each class, then selecting the best fit and applying this class to each person. For each class, we calculated the mean likelihood (min-max) at class-level. This mean likelihood describes how well, on average, a person fits to the class he/she has the highest probability of belonging to. All analyses were conducted using the proc LCA –add-on in SAS statistical software package (SAS, version 9.4, SAS Institute, Cary, NC) [26, 27].

## Results

Table 1 presents the characteristics of the study population, including both the variables that were used in latent class forming the subtypes as well as the variables adjusted for as covariates. Among the total of 1995 participants, two thirds were women (65.3%). The age distribution was similar between men and women. As shown in Table 1, daily use of controller asthma medication was common (78.1%). It is noticeable that, except for the use of inhaled corticosteroids, men and women differed significantly with respect to the use of asthma medications and of health care facilities, even though the mean St. George’s score was similar among the genders (Table 1). COPD diagnosis was more common among men, whereas allergic diseases where more common among women (Table 1).

### Latent classes

We tested all the analyses by applying 2 to 6 classes, and compared the fit-indices between the different models to choose the best fitting classification (see Additional file 1: Table S1) [33, 34]. When two or more models had similar fit-indices, we selected the amount of classes based on clinical interpretability of the results. In order to elaborate all possible combinations of control and severity, we categorized both into four levels: Fully controlled, Controlled, Partly controlled, and Uncontrolled, and Mild, Moderate, Unknown severity, and Severe.

Five classes combining asthma control and severity, were formed for the whole study population: 1) *Fully controlled, mild asthma*, 2) *Partly controlled, mild asthma,* 3) *Partly controlled, moderate asthma,* 4) *Uncontrolled, unknown severity,* and 5) *Uncontrolled, severe asthma.* Further analyses [33] indicated that applying these five subtypes actually masked the differences between men and women, and thus, we further performed sex-specific analyses. For the results of the five-class model and the odds ratios for the predictive factors in these analyses, please refer to the Additional file 1.

Four subtypes of asthma were identified for women (Table 2). Among men the fit-indices were virtually identical for the two and three class model. In such cases, it is recommendable to apply the classification that has better clinical interpretability [26, 27]. Thus, we selected the three class model. The subtypes identified for female asthmatics were the following: 1) *Controlled, mild asthma*, 2) *Partly controlled, moderate asthma*, 3) *Uncontrolled asthma, unknown severity*, and 4) *Uncontrolled, severe asthma*. The corresponding class membership probabilities were 0.41 (0.35–0.46), 0.24 (0.19–0.28), 0.26 (0.20–0.32), and 0.09 (0.06–0.12), as shown in Table 2.

**Table 2:** Subtype analyses according to gender

	Women				Men		
	Mild	Moderate	Unknown severity	Severe	Mild	Unknown severity	Severe
	Controlled asthma	Partly controlled asthma	Uncontrolled asthma	Uncontrolled asthma	Controlled asthma	Uncontrolled asthma	Partly controlled asthma
	Probability (95% CI)	Probability (95% CI)	Probability (95% CI)	Probability (95% CI)	Probability (95% CI)	Probability (95% CI)	Probability (95% CI)
Class membership probabilities	0.41 (0.35–0.46)	0.24 (0.19–0.28)	0.26 (0.20–0.32)	0.09 (0.06–0.12)	0.31 (0.22–0.39)	0.53 (0.44–0.62)	0.17 (0.12–0.21)
Item Response probabilities							
Asthma controller medication^a^							
Not at all	**0.14** (0.11–0.18)	0.02 (0.00–0.03)	0.00 (0.00–0.01)	0.00 (0.00–0.00)	**0.16** (0.09–0.22)	0.04 (0.01–0.06)	0.01 (0.00–0.03)
Occasionally	0.22 (0.18–0.26)	0.12 (0.07–0.17)	0.13 (0.07–0.18)	0.06 (0.01–0.12)	0.20 (0.13–0.27)	0.14 (0.09–0.19)	0.09 (0.02–0.16)
Daily	**0.64** (0.59–0.69)	**0.86** (0.81–0.91)	**0.87** (0.82–0.93)	**0.94** (0.88–0.99)	**0.64** (0.56–0.73)	**0.82** (0.76–0.87)	**0.90** (0.83–0.97)
Bronchodilator use							
Not at all	**0.30** (0.25–0.34)	0.10 (0.05–0.14)	0.10 (0.05–0.14)	0.02 (0.00–0.05)	**0.39** (0.30–0.49)	0.17 (0.12–0.23)	0.05 (0.00–0.11)
Occasionally	**0.67** (0.62–0.72)	**0.75** (0.69–0.82)	**0.59** (0.52–0.67)	**0.57** (0.46–0.68)	**0.61** (0.51–0.70)	**0.54** (0.47–0.62)	0.45 (0.34–0.56)
Daily	0.04 (0.01–0.06)	0.15 (0.09–0.21)	0.31 (0.23–0.38)	**0.49** (0.38–0.60)	0.00 (0.00–0.01)	**0.28** (0.21–0.35)	**0.50** (0.39–0.61)
Oral corticosteroid prescriptions							
0 prescriptions	**0.97** (0.94–0.99)	0.41 (0.32–0.49)	**0.78** (0.71–0.84)	0.11 (0.03–0.18)	**1.00** (0.98–1.00)	**0.90** (0.86–0.95)	0.28 (0.16–0.40)
1 to 2 prescriptions	0.03 (0.00–0.05)	**0.57** (0.49–0.66)	0.15 (0.09–0.21)	**0.53** (0.41–0.65)	0.00 (0.00–0.02)	0.09 (0.04–0.13)	**0.51** (0.39–0.63)
≥3 prescriptions	0.00 (0.00–0.00)	0.02 (0.00–0.05)	0.02 (0.00–0.03)	**0.32** (0.21–0.43)	0.00 (0.00–0.00)	0.01 (0.00–0.02)	0.16 (0.08–0.24)
Daily oral corticosteroid medication	0.01 (0.00–0.02)	0.00 (0.00–0.00)	0.06 (0.03–0.09)	0.04 (0.00–0.09)	0.00 (0.00–0.00)	0.00 (0.00–0.01)	0.05 (0.00–0.10)
Antibiotics use							
0 prescription	**0.98** (0.95–1.00)	**0.42** (0.33–0.52)	**0.97** (0.93–1.01)	0.08 (0.00–0.19)	**0.99** (0.97–1.01)	**0.95** (0.92–0.99)	0.37 (0.23–0.50)
1 prescription	0.01 (0.00–0.03)	**0.32** (0.25–0.40)	0.02 (0.00–0.05)	0.08 (0.00–0.16)	0.01 (0.00–0.03)	0.04 (0.01–0.06)	0.24 (0.14–0.33)
2 prescriptions	0.01 (0.00–0.02)	0.17 (0.11–0.22)	0.01 (0.00–0.03)	0.20 (0.10–0.29)	0.00 (0.00–0.01)	0.01 (0.00–0.02)	0.19 (0.10–0.27)
3 or more prescriptions	0.00 (0.00–0.01)	0.09 (0.04–0.14)	0.00 (0.00–0.03)	**0.64** (0.49–0.78)	0.00 (0.00–0.00)	0.00 (0.00–0.01)	**0.21 (0.12–0.30)**
St. George’s Score^b^							
0–7 points	**0.48** (0.41–0.55)	0.15 (0.10–0.21)	0.03 (0.01–0.05)	0.00 (0.00–0.01)	**0.59** (0.47–0.72)	0.14 (0.08–0.20)	0.07 (0.01–0.13)
>7–15 points	0.32 (0.27–0.38)	0.31 (0.24–0.37)	0.15 (0.13–0.18)	0.09 (0.02–0.16)	0.21 (0.12–0.31)	0.26 (0.20–0.33)	0.04 (0.00–0.09)
>15–27 points	0.15 (0.10–0.21)	**0.35** (0.28–0.42)	0.36 (0.28–0.43)	0.17 (0.08–0.27)	0.19 (0.09–0.28)	0.27 (0.20–0.33)	0.28 (0.18–0.38)
>27 points	0.04 (0.01–0.08)	0.19 (0.11–0.27)	**0.49** (0.39–0.58)	**0.74** (0.63–0.85)	0.00 (0.00–0.03)	**0.33** (0.26–0.41)	**0.62** (0.51–0.73)
Health care facility use score							
0 point	**0.97** (0.94–0.99)	0.36 (0.27–0.45)	**0.88** (0.82–0.94)	0.13 (0.04–0.22)	**0.96** (0.92–1.00)	**0.92** (0.87–0.97)	**0.37** (0.26–0.49)
1 point	0.03 (0.01–0.06)	0.21 (0.15–0.27)	0.07 (0.03–0.11)	0.03 (0.00–0.07)	0.04 (0.01–0.08)	0.03 (0.00–0.06)	0.12 (0.05–0.19)
2 to 3 points	0.00 (0.00–0.00)	**0.38** (0.30–0.46)	0.05 (0.01–0.09)	**0.48** (0.37–0.59)	0.00 (0.00–0.01)	0.05 (0.01–0.08)	0.34 (0.24–0.44)
≥4 points	0.00 (0.00–0.00)	0.05 (0.02–0.08)	0.00 (0.00–0.02)	**0.36** (0.25–0.47)	0.00 (0.00–0.00)	0.00 (0.00–0.00)	0.17 (0.09–0.25)

Probabilities over 50% or otherwise important for the characterization of each subtype are marked in bold

^a^Asthma controller medication use includes both inhaled corticosteroids and combination medication of inhaled corticosteroid and long-acting beta-agonist

^b^St. George’s score range: 0-100

*Controlled, mild asthma* was characterized by a probability of 0.14 (0.11–0.18) for using no ICS, in combination with the largest proportion of subjects needing no bronchodilators, oral corticosteroids, or antibiotics, and low use of health-care. As much as 0.48 (0.41–0.55) of subjects in this subtype had a SGTS at the healthy person’s level.

Among the *Partly controlled, moderate asthma* subtype most subjects (86%) used daily ICS (Table 2). In addition, occasional BD use was in this group the highest of all classes (0.75, 0.69–0.82). They reported some use of AB and OCS. Healthcare use was heterogeneous among this class (Table 2). SGTS was mostly above the healthy person’s level.

*Uncontrolled asthma, unknown severity* was described by no health-care use, no AB or OCS, despite 0.49 (0.39–0.58) of the subjects had SGTS above 27 indicating poor control.

In the *Uncontrolled, severe asthma* subtype the proportion of subjects using regular ICS was high at 0.94 (0.88–0.99) and also their use of daily bronchodilator medication (BD) was high, as was their use of OCS and AB as well as HCU. The majority of this subtype, i.e. 0.74 (0.63–0.85), had SGTS at the highest level indicating the poorest asthma control, while no subject was at the healthy person’s level.

Among men the identified subtypes differed substantially from those identified among women, since the use of asthma medications and healthcare services were low despite of high SGTS indicating poor asthma control. Use of BD was large among all asthma subtypes among men. The subtypes identified were: 1) *Controlled, mild asthma*, 2) *Uncontrolled, unknown severity,* and 3) *Partly controlled, severe asthma*.

*Controlled, mild asthma* was characterized with SGTS-scores at the healthy person’s level (0.59, 0.47–0.72) indicating good asthma control, with no use of oral medications or need for HCU (see Table 2).

For *Uncontrolled, unknown severity* the distribution of SGTS was wide (Table 2). The probability of this group using daily inhaled corticosteroids (ICS) was high at 0.82 (0.76–0.87), but using daily BD was also common. Despite being poorly controlled, this class used OCS rarely and reported low HCU (Table 2).

For *Partly controlled, severe asthma* the mean SGTS was high (indicating poor control), although the percentage of daily users of ICS was high at 90%. Use of daily BD was also high, and for this group, the use of OCS and AB was also common suggesting severe asthma in combination with high HCU scores (Table 2).

The best individual mean posterior probability for each class among women and men are displayed in Table 3. Only 37 women (2.84%) and 1 man (0.14%) had this best posterior probability below 0.50. This indicates that if we wish to apply these subtypes into clinical practice, taking into account the gender of the patient is needed to make this classification accurate and useful at the patient level.

**Table 3:** Best fitting posterior probabilities in each class in the crude and adjusted models

	Crude model (adjusted for gender^a^ and age only)	Adjusted model
	Mean (minimum value – maximum value)	Mean (minimum value – maximum value)
The whole population		
Fully controlled, Mild asthma	0.64 (0.35–0.95)	0.82 (0.37–1.00)
Partly Controlled, Mild asthma	0.83 (0.36–1.00)	0.83 (0.43–1.00)
Partly controlled, Moderate asthma	0.77 (0.42–1.00)	0.77 (0.35–1.00)
Uncontrolled asthma, Unknown severity	0.79 (0.42–1.00)	0.83 (0.31–1.00)
Uncontrolled, Severe asthma	0.77 (0.34–0.96)	0.71 (0.36–0.99)
No. (%) of people below 0.50	187 (9.37)	108 (5.76)
Women		
Controlled, Mild asthma	0.74 (0.37–1.00)	0.79 (0.34–1.00)
Partly controlled, Moderate asthma	0.85 (0.39–1.00)	0.88 (0.39–1.00)
Uncontrolled asthma, Unknown severity	0.88 (0.41–1.00)	0.81 (0.39–1.00)
Uncontrolled, Severe asthma	0.88 (0.39–1.00)	0.88 (0.45–1.00)
No. (%) of people below 0.50	37 (2.84)	53 (4.31)
Men		
Controlled, Mild asthma	0.81 (0.53–0.99)	0.90 (0.49–1.00)
Uncontrolled asthma, Unknown severity	0.81 (0.50–1.00)	0.82 (0.44–1.00)
Partly controlled, Severe asthma	0.90 (0.40–1.00)	0.92 (0.40–1.00)
No. (%) of people below 0.50	1 (0.14)	10 (1.55)

^a^Gender not adjusted for in the subtype analyses of women and men

### Factors predicting latent class membership

In the LCA, the different subtypes of asthma are kept fixed when covariates are included in the model. The purpose of adding the covariates is to evaluate whether individuals’ probability to belong to the studied asthma subtypes changes. Thus, the posterior probabilities for each individual need to be recalculated, while the item-response probabilities do not. For each covariate, we calculated the Likelihood *X* ^2^-ratio *P*-value, which indicates whether the corresponding variable is a good predictor of class membership (see Table 4).

**Table 4:** Factors predicting class membership^a^, i.e. asthma subtype, among women and men separately

	Women				Men		
	Moderate Partly controlled asthma	Unknown severity Uncontrolled asthma	Severe Uncontrolled asthma		Unknown severity Uncontrolled asthma	Severe Partly controlled asthma	
	Odds ratio (95% CI)	Odds ratio (95% CI)	Odds ratio (95% CI)	LR test *P*-value^b^	Odds ratio (95% CI)	Odds ratio (95% CI)	LR test *P*-value^a^
Age							
<30	Reference				Reference		
30–59	1.49 (0.88–2.52)	*8.88 (1.37–57.58)*	2.02 (0.77–5.26)	0.00	*3.35 (1.10–10.18)*	*4.10 (1.14–14.72)*	0.00
≥60	1.76 (0.90–3.44)	*22.25 (3.30–149.98)*	2.01 (0.68–5.96)	0.00	*5.32 (1.64–17.28)*	*4.84 (1.25–18.77)*	0.00
BMI							
≤25	Reference				Reference		
26–30	1.12 (0.74–1.68)	1.28 (0.72–2.28)	1.42 (0.77–2.61)	0.69	1.09 (0.55–2.15)	1.89 (0.93–3.83)	0.27
>30	1.46 (0.85–2.49)	*4.34 (2.53–7.44)*	*3.20 (1.71–5.98)*	0.00	*4.60 (2.26–9.36)*	*4.53 (2.07–9.90)*	0.00
Allergic diseases							
No	Reference				Reference		
Yes	1.23 (0.83–1.82)	1.19 (0.74–1.92)	*1.84 (1.03–3.28)*	0.19	0.93 (0.54–1.62)	1.67 (0.94–2.96)	0.20
COPD diagnosed by a doctor							
No	Reference				Reference		
Yes	1.98 (0.80–4.91)	*7.17 (3.42–15.00)*	*10.74 (4.83–23.88)*	0.00	*9.01 (4.35–18.66)*	*9.57 (4.62–19.83)*	0.00

^a^Controlled, mild asthma was the reference asthma subtype

^b^Likelihood *X* ^2^-ratio *P*-value

Italicized Odds Ratio are statistically significant at *P*-value < 0.05

The results of the covariate analyses are displayed in Table 4. For both genders *Controlled, mild asthma* was used as the reference asthma category, thus this category is not shown in Table 4. Among women, age over 60 was a strong predictor for *Uncontrolled asthma with unknown severity* (OR: 22.25, 95% CI: 3.30–149.98). In addition, subjects with the age of 30 to 59 years showed a significantly elevated odds ratio for this subtype membership. In addition, subjects with BMI > 30 showed a high odds ratio for this subtype among women (OR: 7.17, 95% CI: 3.42–15.00). Allergic diseases predicted *Uncontrolled, severe asthma* subtype membership with an odds ratio of 1.84 (1.03–3.28).

Among men, identifying separate predictors for *Partly controlled, severe asthma* and *Uncontrolled asthma with unknown severity* was difficult, as almost all factors predicted belonging to these subtypes with similar odds ratios (Table 4). The only exception to this trend was that those who reported having allergic diseases showed a higher odds ratio for belonging to the *Partly controlled, severe asthma* subtype (1.67, 0.94–2.96) compared to the *Uncontrolled asthma with unknown severity* subtype (0.93, 0.54–1.62). COPD was a significant predictor for both *Poorly controlled asthma with unknown severity* and *Partly controlled, severe asthma* subtypes with the corresponding odds ratios of 9.01 (4.35–18.66) and 9.57 (4.62–19.83).

When adjusting for all covariates, the best posterior probabilities calculated among men and women were elevated compared to the non-adjusted model (Table 3). In this analysis, 53 women (4.31%) and 10 men (1.55%) had their best posterior probability below 0.50. On the other hand, the mean best posterior probability for each asthma subtype improved or remained stable, indicating that for those assigned to that class, the accuracy was better, although there were more people wavering between subtypes (Table 3). This is most likely due to the fact, that the optimal set of covariates is different between men and women.

## Discussion

This population-based cross-sectional study provides evidence that identifying clinically meaningful subtypes of asthma based on questionnaire data is possible. The classification above benefits clinical work, because it provides a simpler way of categorizing asthmatics without using complicated clinical measurements. We performed the subtype analyses among women and men separately and found that gender-specific analysis is essential when assessing asthma control and severity. We also show that by calculating the class membership probability, the results of latent class analyses become easier to interpret and apply, because each individual patient can now be placed to one of the subtypes. We found high posterior probabilities which show that most individuals fit well into their respective subtype. This method, although quite informative, has to our knowledge been applied in only one previous study on asthma subtypes [10].

The finding that, especially among men, there is a large group of asthmatics with insufficient use of medication and healthcare services is worrying, and it calls for better patient-education programs. It is noticeable that among women higher age predicts the risk of uncontrolled asthma, but that it is also linked to poorer use of medical services. Thus, older age seems to create a difficulty when assessing the severity of asthma among women. Obese people were more likely to belong to the subtypes of asthma with severe disease and poor asthma control among both women and men. Among men, predictors separating subtypes of severe manifestations and those of unknown severity could not be identified. However, concomitant COPD was linked with severe manifestations of asthma, irrespective of the asthma subtype. For clinical work it is important to recognize the subtypes at risk of poorer asthma management since it might be possible to regain better control of their disease and thus prevent future complications of poorly managed disease [23, 24].

### Validity of results

The previous studies that have applied similar type of analysis methods, have included factors for classification that were a mixture of etiological factors, outcomes of asthma, characteristics of individuals, and presence of atopic manifestations [1–12]. In this study, we included in the subtype analyses only factors that are related to the manifestations or treatment of asthma. Thus, our approach provides a new alternative method for identifying subtypes of asthma that is more easily applicable for clinical work, especially in primary care settings with limited resources. The strengths of our study include the fact that asthma diagnosis was based on registry data received from the Social Insurance Institution of Finland. The criteria that they require for warranting the reimbursement right for asthma medication are clearly defined in Finland and follow the criteria recommended by the national asthma program report [35, 36]. Thus, the diagnosis of asthma was accurate and consistent between different asthma patients in this study. The questionnaires were distributed by The Social Insurance Institution of Finland. This was because, under the current law, the investigators are not allowed to contact directly asthmatics identified through this registry. This explains why the response rate (40%) was satisfactory, but lower than in our other epidemiologic studies [37, 38]. We have many subjects (26% in women and 53% in men) whose asthma severity is defined as “unknown”. This is mainly a question of labelling. We could have called this subtype moderate severity, but since the ATS/ERS guidelines require assessment of asthma severity under “adequate treatment” [25], we cannot fully assess the severity of their asthma, as this group does not seem to use all the treatments that are available. Introduction of some degree of information bias due to over-reporting of symptoms is possible in this study. However, a bias would be introduced only if such over-reporting would be related to the determinants of the subtype that the individual belongs to.

We did not verify the subtypes with full scale lung function testing and were not able to verify the results in another population due to the uniqueness of this data collection, these questions remain for future studies. This is also the situation often faced in clinical work, especially in primary care, as well as in large epidemiologic studies. We also did not have the possibility to test the population for biomarkers and form endotypes out of this population, which would be the next step towards investigating whether these subtypes might influence asthma treatment in the future [39]. It is however highly likely, that behind the observed subtypes there are underlying mechanisms, for example inflammation that at least partly would explain the differences in asthma control and severity [39].

### Synthesis with previous knowledge

As the choice of the variables applied in our study is in many ways different compared to previous studies that have applied similar type of analysis methods, a comparison between the results is difficult [1–12]. However, we did identify some subtypes that are similar to those found in other populations. For example, our subtype *Uncontrolled, Severe asthma* among women is comparable to groups called “severe” or “difficult-to treat” asthma in some previous studies [1, 4]. However, we did not find such subtype among men, as in no class was the asthma-related medication or health-care use as extensive as among women belonging to this class. In addition, our subtype of *Controlled, mild asthma* is comparable to the one called “inactive/mild untreated adult onset asthma” in previous studies [4]. According to our literature review, no previous study has examined asthma subtypes among men and women separately in stratified analyses. According to our findings this is an essential feature in the analyses, since especially the assessment of asthma severity seems to differ substantially between the genders in clinical practice.

We included potential determinants of class membership, i.e. asthma subtype, in our analyses, such as COPD [21], allergic diseases [20], age [22], and obesity [19]. In our study, all of these predicted membership of subtypes with poor control and increased severity. However, the posterior probability of the best-fitting class did not improve after adding these covariates, which indicates that the optimal set of covariates may differ between men and women.

## Conclusions

This population-based cross-sectional study provides evidence that adult asthma can be classified into subtypes according to the level of asthma control and asthma severity based on questionnaire-derived variables. We identified asthma subtypes separately among men and women which was crucial for the accuracy of the classification. We used latent class analyses to subtype asthmatics. In addition, we identified several predictors for subtypes that are characterized by poor asthma control and severe disease. We applied the capacity of LCA to estimate an individual’s probability to belong to the formed subtype of asthma. Our results show that most individuals can be placed accurately into the subtypes identified. In the future, such subtyping may facilitate treatment of asthma patients in primary care.

## Additional file

Additional file 1: The subtypes of asthma among the whole study population. (DOCX 35 kb)
