# Supplementary material for: Subtypes of asthma based on asthma control and severity: a latent class analysis
Source: Respir Res. 2017 Jan 23;18:24. doi: 10.1186/s12931-017-0508-y (PMC5259948; doi:10.1186/s12931-017-0508-y)
Supplement: Additional file 1: — The subtypes of asthma among the whole study population. (DOCX 35 kb) [file 12931_2017_508_MOESM1_ESM.docx]

**Mäkikyrö EMS, Jaakkola MS, Jaakkola JJK. Subtypes of asthma based on asthma control and severity: a latent class analysis – Supplementary material**

**The subtypes of asthma among the whole study population**

For the whole study population, the five class model was the best fitting (Table S1). Further analyses indicated that men and women should be analyzed separately, so that is why we present the analyses among the whole population only here. The five class (i.e. five subtypes) model showed the best fit. The classes were named: 1) *Fully controlled, mild asthma*, 2) *Controlled, mild asthma,* 3) *Partly controlled, moderate asthma,* 4) *Uncontrolled asthma, unknown severity,* and 5) *Uncontrolled, severe asthma.* The corresponding class membership probabilities were 1) 0.22 (95% CI: 0.14-0.29), 2) 0.26 (0.20-0.33), 3) 0.16 (0.13-0.20), 4) 0.27 (0.18-0.36), and 5) 0.09 (0.06-0.11). The best fitting class posterior probability means (min-max) were: 1) 0.64 (0.35-0.95), 2) 0.83 (0.36-1.00), 3) 0.77 (0.42-1.00), 4) 0.79 0.42-1.00), and 5) 0.77 (0.34-0.96). Only 187 subjects (9.37%) had posterior probabilities below 0.50, this indicates that when a person was assigned to a certain class, he/she was also likely belong to it (Table 4).

*Fully controlled, mild asthma* was characterized by the least use of ICS and BD, and no use of AB or OCS. St. George’s respiratory scores (SGTS) were mostly at the healthy person’s level. *Controlled, mild asthma* was also described by low SGTS, but more ICS and BD use, while still no HCU or use of oral medication. *Partly controlled, moderate asthma* was described by some oral medications, intermediate HCU scores, and higher use of daily ICS (0.86, 0.81-0.91) and BD (0.12, 0.06-0.13). *Uncontrolled asthma with unknown severity* was characterized by low BD and ICS use, low HCU scores, and low OCS and AB use, despite of higher SGTS (0.44, 0.37-0.50, scores over 27). This group has at the moment severe manifestations, but due to poor compliance with asthma medications their “true” severity cannot be estimated. Subjects in *Uncontrolled, severe asthma* had the highest SGTS, but also most ICS, BD, OCS and AB use and high HCU. (Table S2)

The results of the analyses for the predicting factors are displayed in Table S3. Age was a significant predictor of all other subtypes except *Partly controlled, moderate asthma.* For those above 60 years, the odds for *Uncontrolled asthma, unknown severity* is 8.62 (95% CI: 4.00-18.57), but the odds ratio was also elevated for *Controlled, mild asthma* (OR 7.98, 2.23-28.50) indicating that some elderly people have their asthma in good control and that ageing does not directly cause poor asthma control. Obesity predicts membership of classes with poorer asthma control and poorer compliance. The highest odds was for *Uncontrolled, severe asthma* (5.03, 2.69-9.41), but *Partly controlled, moderate asthma* and *Uncontrolled asthma, unknown severity* had also high odds ratios, whereas for *Controlled, mild asthma* the odds ratio for BMI over 30 was low (0.22, 0.04-1.06). Those with allergic diseases were most likely to belong to the classes receiving active medical attention. The odds ratio was for *Partly controlled, moderate asthma* 1.65 (1.06-2.56) and for *Uncontrolled, severe asthma* 2.19 (1.20-3.99). COPD-diagnosis was a predictor for the classes with poorer asthma control and severe or unknown severity of disease. For *Uncontrolled, severe asthma* the predictor odds ratio was as high as 21.61 (10.79-43.26). (Table S3)

| **Table S1**. **Fit Statistics Indicating Best Model Fit** | | | | | | | | | |
| --- | --- | --- | --- | --- | --- | --- | --- | --- | --- |
| **No. of groups** |  | **Log-likelihood** | **G-squared** | **AIC** | **BIC** | **CAIC** | **a-BIC** | **Entropy** | **DF** |
| **Whole study population** | | | | | | | | | |
| 2 |  | -9483.39 | 1154.10 | 1220.10 | 1404.84 | 1437.84 | 1300.00 | 0.77 | 2270 |
| 3 |  | -9408.56 | 1004.43 | 1104.43 | 1384.35 | 1434.35 | 1225.50 | 0.72 | 2253 |
| 4 |  | -9326.46 | 840.23 | 974.23 | **1349.32** | **1416.32** | 1136.46 | 0.62 | 2236 |
| **5** |  | -9288.66 | 764.63 | 932.63 | 1402.89 | 1486.89 | **1136.02** | 0.60 | 2219 |
| 6 |  | -9268.57 | **724.45** | **926.45** | 1491.89 | 1592.89 | 1171.01 | 0.61 | 2202 |
| **Women** | | | | | | | | | |
| 2 |  | -6407.67 | 987.94 | 1053.94 | 1224.63 | 1257.63 | 1119.80 | 0.76 | 2270 |
| 3 |  | -6335.24 | 843.08 | 943.08 | 1201.70 | **1251.70** | 1042.88 | 0.72 | 2253 |
| **4** |  | -6274.08 | 720.77 | 854.77 | **1201.32** | 1268.32 | **988.50** | 0.66 | 2236 |
| 5 |  | -6251.82 | 676.24 | 844.24 | 1278.72 | 1362.72 | 1011.89 | 0.68 | 2219 |
| 6 |  | -6225.79 | **624.18** | **826.18** | 1348.59 | 1449.59 | 1027.76 | 0.69 | 2202 |
| **Men** | | | | | | | | | |
| 2 |  | -3012.81 | 530.09 | 596.09 | **745.90** | **778.90** | **641.12** | 0.79 | 2270 |
| **3** |  | -2986.17 | 476.80 | 576.80 | 803.78 | 853.78 | 645.03 | 0.84 | 2253 |
| 4 |  | -2960.67 | 425.81 | 559.81 | 863.96 | 930.96 | 651.22 | 0.71 | 2236 |
| 5 |  | -2947.89 | 400.24 | 568.24 | 949.57 | 1033.57 | 682.86 | 0.63 | 2219 |
| 6 |  | -2924.36 | **353.18** | **555.18** | 1013.68 | 1114.68 | 692.99 | 0.72 | 2202 |
| The fit indices in bold indicate best model fit. The best model has been chosen according to the adjusted Bayesian information criterion value and interpretability | | | | | | | | | |

| **Table S2. Results of the Five Class (Subtype) Model for the Whole Study Population** | | | | | | | | |
| --- | --- | --- | --- | --- | --- | --- | --- | --- |
|  | **Mild** | | **Moderate** | **Unknown severity** | | | | **Severe** |
|  | **Fully controlled asthma** | **Controlled asthma** | **Partly controlled asthma** | **Uncontrolled asthma** | | **Uncontrolled asthma** | | |
|  | **N (95% CI)** | **N (95% CI)** | **N (95% CI)** | **N (95% CI)** | | **N (95% CI)** | | |
| **Class membership**  **probabilities** | 0.22 (0.14-0.29) | 0.26 (0.20-0.33) | 0.16 (0.13-0.20) | 0.27 (0.18-0.36) | | 0.09 (0.06-0.11) | | |
| **Item Response probabilities** | | | | | | | | |
| **Controller asthma medication*** | | | | | | | | |
| Not at all | **0.23 (0.18-0.29)** | 0.01 (0.00-0.04) | 0.01 (0.00-0.03) | 0.03 (0.01-0.05) | | 0.01 (0.00-0.02) | | |
| Occasionally | 0.12 (0.07-0.17) | **0.35 (0.28-0.43)** | 0.12 (0.08-0.17) | 0.08 (0.04-0.13) | | 0.06 (0.01-0.11) | | |
| Daily | **0.64 (0.57-0.71)** | **0.64 (0.56-0.71)** | **0.86 (0.81-0.91)** | **0.88 (0.84-0.93)** | | **0.93 (0.88-0.98)** | | |
| **Bronchodilator use** | | | | | | | | |
| Not at all | **0.53 (0.43-0.63)** | 0.01 (0.00-0.06) | 0.10 (0.06-0.15) | 0.17 (0.13-0.22) | | 0.02 (0.00-0.05) | | |
| Occasionally | 0.44 (0.34-0.54) | **0.96 (0.88-1.00)** | **0.77 (0.70-0.84)** | **0.48 (0.41-0.56)** | | 0.46 (0.36-0.56) | | |
| Daily | 0.03 (0.00-0.06) | 0.03 (0.00-0.09) | 0.12 (0.06-0.13) | 0.34 (0.28-0.41) | | **0.52 (0.42-0.62)** | | |
| **Oral corticosteroid prescriptions** | | | | | | | | |
| 0 prescriptions | **0.98 (0.96-1.00)** | **0.94 (0.89-1.00)** | 0.39 (0.30-0.47) | **0.83 (0.77-0.88)** | | 0.14 (0.07-0.21) | | |
| 1 to 2 prescriptions | 0.01 (0.00-0.03) | 0.05 (0.01-0.09) | **0.59 (0.51-0.67)** | 0.12 (0.08-0.17) | | **0.51 (0.41-0.61)** | | |
| >= 3 prescriptions | 0.00 (0.00-0.00) | 0.00 (0.00-0.02) | 0.02 (0.00-0.05) | 0.01 (0.00-0.02) | | **0.30 (0.21-0.39)** | | |
| Daily corticosteroid | 0.01 (0.00-0.02) | 0.00 (0.00-0.03) | 0.00 (0.00-0.02) | 0.04 (0.02-0.06) | | 0.05 (0.01-0.09) | | |
| **Antibiotics use** | | | | | | | | |
| 0 prescription | **0.98 (0.96-1.00)** | **0.98 (0.96-1.00)** | **0.38 (0.28-0.47)** | **0.94 (0.91-0.97)** | | 0.21 (0.11-0.31) | | |
| 1 prescription | 0.02 (0.00-0.04) | 0.00 (0.00-0.03) | **0.33 (0.26-0.40)** | 0.04 (0.02-0.07) | | 0.12 (0.05-0.19) | | |
| 2 prescriptions | 0.00 (0.00-0.00) | 0.00 (0.00-0.02) | 0.19 (0.13-0.25) | 0.01 (0.00-0.03) | | 0.17 (0.10-0.25) | | |
| 3 or more prescriptions | 0.00 (0.00-0.00) | 0.01 (0.00-0.02) | 0.10 (0.05-0.15) | 0.01 (0.00-0.02) | | **0.50 (0.39-0.61)** | | |
| **St. George’s Score** † | | | | | | | | |
| 0-7 points | **0.63 (0.56-0.71)** | **0.30 (0.21-0.39)** | 0.15 (0.10-0.20) | 0.07 (0.02-0.12) | | 0.00 (0.00-0.01) | | |
| >7-15 points | 0.25 (0.19-0.32) | **0.37 (0.30-0.44)** | 0.29 (0.22-0.36) | 0.17 (0.11-0.22) | | 0.05 (0.00-0.10) | | |
| >15-27 points | 0.11 (0.06-0.16) | 0.23 (0.16-0.30) | **0.36 (0.29-0.43)** | **0.33 (0.28-0.38)** | | 0.19 (0.11-0.27) | | |
| >27 points | 0.00 (0.00-0.01) | 0.10 (0.04-0.15) | 0.20 (0.12-0.29) | **0.44 (0.37-0.50)** | | **0.76 (0.67-0.85)** | | |
| **Health care facility use score** | | | | | | | | |
| 0 points | **0.99 (0.98-1.00)** | **0.89 (0.85-0.94)** | 0.33 (0.24-0.42) | | **0.91 (0.87-0.95)** | | 0.23 (0.14-0.32) | |
| 1 point | 0.01 (0.00-0.02) | 0.08 (0.04-0.11) | 0.21 (0.15-0.27) | | 0.05 (0.02-0.07) | | 0.05 (0.00-0.10) | |
| 2 to 3 points | 0.00 (0.00-0.00) | 0.03 (0.00-0.05) | **0.39 (0.31-0.47)** | | 0.04 (0.02-0.07) | | **0.43 (0.33-0.52)** | |
| >= 4 points | 0.00 (0.00-0.00) | 0.00 (0.00-0.01) | 0.07 (0.03-0.10) | | 0.00 (0.00-0.00) | | **0.30 (0.21-0.39)** | |
| *Inhaled corticosteroids and combination medication (long-acting beta-agonist and corticosteroid) included as controller medications  †St. George’s total score (SGTS) range 0-100 | | | | | | | | |

| **Table S3. Factors Predicting Class (i.e. Subtype) Membership in the Five-Class Model when using *Mild, Fully Controlled Asthma* as the Reference Class** | | | | | |
| --- | --- | --- | --- | --- | --- |
|  | **Mild** | **Moderate** | **Unknown severity** | **Severe** |  |
|  | Controlled asthma | Partly controlled asthma | Uncontrolled asthma | Uncontrolled asthma |  |
|  | **Odds ratio (95% CI)** | **Odds ratio (95% CI)** | **Odds ratio (95% CI)** | **Odds ratio (95% CI)** | **LR test P-value*** |
| ***Gender*** | | | | | |
| Male | Reference | Reference | Reference | Reference |  |
| Female | 1.68 (0.84-3.36) | **5.30 (2.85-9.83)** | 1.16 (0.82-1.64) | 1.65 (0.96-2.85) | 0.00 |
| ***Age*** | | | | | |
| < 30 | Reference | Reference | Reference | Reference |  |
| 30-59 | **4.74 (1.53-14.72)** | 1.59 (0.92-2.71) | **4.05 (1.99-8.22)** | **3.92 (1.08-14.30)** | 0.00 |
| ≥ 60 | **7.98 (2.23-28.50)** | 1.44 (0.68-3.04) | **8.62 (4.00-18.57)** | **5.30 (1.22-23.08)** | 0.00 |
| ***BMI*** | | | | | |
| ≤ 25 | Reference | Reference | Reference | Reference |  |
| 26-30 | 1.05 (0.60-1.84) | 1.31 (0.75-2.30) | 1.41 (0.94-2.12) | 1.87 (0.98-3.58) | 0.20 |
| >30 | 0.22 (0.04-1.06) | **2.47 (1.20-5.06)** | **4.39 (2.86-6.73)** | **5.03 (2.69-9.41)** | 0.00 |
| ***Allergic diseases*** | | | | | |
| No | Reference | Reference | Reference | Reference |  |
| Yes | 0.71 (0.33-1.52) | **1.65 (1.06-2.56)** | 1.12 (0.79-1.59) | **2.19 (1.20-3.99)** | 0.00 |
| ***COPD diagnosed by a doctor*** | | | | | |
| No | Reference | Reference | Reference | Reference |  |
| Yes | 0.53 (0.10-2.90) | **3.00 (1.12-8.03)** | **8.51 (4.81-15.07)** | **21.61 (10.79-43.26)** | 0.00 |
| * Likelihood X^2^-ratio P-value  Class Fully controlled, mild asthma not displayed in the table due to it acting as the reference class for asthma. | | | | | |
